# Support not corresponding to transition to a new treatment: Women's perceptions of support provided by their male partners during hormonal therapy

**DOI:** 10.3402/qhw.v10.29283

**Published:** 2015-11-30

**Authors:** Sena Yamamoto, Keiko Tazumi, Harue Arao

**Affiliations:** 1 Division of Health Sciences, Osaka University Graduate School of Medicine, Osaka, Japan; 2 Osaka University Hospital, Osaka, Japan

**Keywords:** Social support, symptom management, psychological health, cancer nursing, content analysis

## Abstract

Women with breast cancer receive support from their partners to deal with the side effects of therapies over the cancer trajectory. Hormonal therapy (HT) is usually given after completing other treatments, and women receiving HT reclaim their normal life. This may lead to changes in support from their partners. Therefore, we explored women's perceptions of the support provided by their male partners in managing the side effects of adjuvant HT. We conducted semi-structured interviews with 10 women who received HT and recognized their partners as a main source of support. An interview guide was used to explore their experiences of treatment side effects, the contents of support received from their partners, their need for support, and their overall relationship with their partners. Interviews were analysed by content analysis. A theme on how participants perceived support from their partners was formulated as “Support not corresponding to transition to a new treatment” with the following categories: “Shrinking support,” “Primacy of partner,” and “Solitary new treatment.” Participants felt lack of support from their partners because their partners did not understand their experience of the side effects induced by HT. Unlike the side effects of past treatments such as surgery and chemotherapy, side effects of HT cannot be observed and are highly subjective. Their partners often failed to notice these symptoms and provided little support. Nevertheless, participants aimed to accept the existing support without asking for more. They were left alone in the continuing trajectory of breast cancer. After starting HT, women entered a new treatment phase in which less understanding and support was provided by partners. Educational support for couples may enable sharing of subjective symptoms that are not obvious to partners and improve outcomes by facilitating partner engagement and support.

Breast cancer is the most common cancer among women worldwide (Bray, McCarron, & Parkin, 2004). Women with breast cancer receive multimodality treatment, including surgery, chemotherapy, radiation therapy, and hormonal therapy (HT). HT as an adjuvant therapy is usually given after completing other treatments and improves the survival of many women with breast cancer. It is useful in the treatment of hormone-receptor-positive breast cancer, which accounts for approximately 60–75% of all invasive breast cancers (Burstein et al., 2014). Adjuvant HT has two main strategies: one uses tamoxifen to block the effects of oestrogen, which stimulates the growth of breast cancer cells; whereas the other uses luteinizing hormone-releasing hormone agonists or aromatase inhibitors to block oestrogen production. These medications are prescribed for long periods, and adjuvant HT has been typically provided for more than 5 years. Recent research has shown that HT for up to 10 years can further reduce the risk of breast cancer recurrence and ultimately death (Burstein et al., 2014).

Despite its success in improving outcomes, HT is not without its problems. Women receiving HT frequently experience a variety of treatment-related side effects, including vasomotor, somatic, sexual, and psychological symptoms (Van Londen et al., 2014; Vincent, 2015). These symptoms are reported to occur with higher prevalence and with greater intensity in women receiving HT than in women with no history of cancer and in women not undergoing HT (Harris, Remington, Trentham-Dietz, Allen, & Newcomb, 2002; Mortimer & Behrendt, 2013). Moreover, these symptoms can remain bothersome throughout the course of the treatment (Mortimer & Behrendt, 2013; Schmid-Büchi, Halfens, Dassen, & Van den Borne, 2008). Therefore, the quality of life for women receiving HT can be negatively affected by these symptoms (Ribi et al., 2007; Van Londen et al., 2014).

Women receiving HT must manage the various side effects that are persistent during long-term treatment; however, as a result of few effective strategies available, they often find this difficult (Van Londen et al., 2014). To compound matters, most of this management has usually been provided without medical support because women receiving HT are treated as outpatients with limited opportunity to receive medical and nursing care.

Social support plays a key role in the management of side effects when contact with healthcare providers is limited. Perceived support has also been shown to influence adaptation to chemotherapy-induced premature menopause, which causes symptoms that are similar to the side effects of HT. Women who adapt to this premature menopause positively have been shown to perceive that they receive strong support (Knobf, 2008). Nevertheless, women receiving HT can be discontented with the lack of understanding shown by family and friends (Van Londen et al., 2014).

Accessing social support is essential and can be the primary support for women during HT. In particular, partners are often identified as the major source of such support over the cancer trajectory (Carlson, Ottenbreit, St Pierre, & Bultz, 2001; Hodgkinson et al., 2007; Kinsinger, Laurenceau, Carver, & Antoni, 2011; Lim, Shon, Paek, & Daly, 2014). Several studies have reported that support from partners can help with successful adaptation to breast cancer and can improve the psychological well-being of women (Bloom, Stewart, Johnston, Banks, & Fobair, 2001; Carver, Smith, Petronis, & Antoni, 2006; Pistrang & Barker, 1995). A study has also identified that psychological interventions have potential benefits for couples with breast cancer (Brandão, Schulz, & Matos, 2014). Partners are the significant source of symptom relief (Regan et al., 2012), and facilitating such support could lead to better management of the side effects of HT.

In this study, we aimed to explore their perceptions of the support provided by partners to manage HT-induced side effects.

## Methods

### Design

We chose a qualitative design using content analysis to attain a condensed and broad description of the phenomenon (Elo & Kyngäs, 2008). Content analysis is described as “a research technique for making replicable and valid inferences from texts (or other meaningful matter) to the contexts of their use” (Krippendorff, 2004, p. 18). It enables us to deal with both manifest and latent content; that is, what the text says as well as what the text is talking about (Graneheim & Lundman, 2004). A characteristic of qualitative content analysis is to emphasize differences and similarities among data (Graneheim & Lundman, 2004). Therefore, we considered it suitable for this study as we aimed to explore women's perceptions of partners’ support during HT, which although it may differ from woman to woman, may also involve common and comprehensive content.

### Participants

We recruited participants from a designated cancer care hospital in Japan. Participants were required to be female, have a diagnosis of primary breast cancer, have received adjuvant HT for at least 3 months, recognize their partners as their main source of social support, live with their partners, and be aged ≥20 years. Partner was defined as a man who had an intimate relationship with the participant, regardless of marital status. Participants’ exclusion criteria were exhibiting a distant metastasis, another type of cancer, a serious comorbidity or severe mental distress, and care for their partners. Ten invited participants completed interviews. The participants were aged between 32 and 65 years. The age of their partners ranged from 35 to 74 years; notably, all partners were spouses. Four participants and eight partners were working. Seven participants lived with their children; two of them also lived with other family members (grandchildren and parents, respectively). The participants had received HT for 3–19 months. Half of them were taking tamoxifen, and the others were taking aromatase inhibitors. All participants underwent surgery and chemotherapy. In addition, six participants were treated with radiation and trastuzumab, two with radiation, and two with trastuzumab.

### Data collection

Potential participants were initially approached by their usual doctors or nurses. Women who indicated interest in participation were contacted by the first author. The first author conducted individual, semi-structured interviews between April and June 2014. All interviews were recorded with the consent of each participant. Interviews lasted for 36.3 min on average (range, 29–41 min).

Questions contained in the interview guide were: “How did your physical and mental condition change after starting HT?,” “How have these changes affected your daily lives?,” “What is the support received from partners in relation to these changes?,” “Please let me know if you have more need for support from your partner,” and “How do you feel about the overall relationship with your partner after the start of HT?” Some open-ended questions were included as additional questions to probe deeper content.

### Data analysis

Data were systematically analysed by content analysis guided by the techniques set out by Graneheim and Lundman (2004). The first author transcribed all recorded interviews verbatim and read the transcripts several times to obtain a general sense of the data. Then, after rereading the transcript, segments that related to the research aim were identified and extracted as the meaning units. These meaning units were then condensed, abstracted, and coded. In turn, subcategories and categories, which constituted the manifest content, were developed by comparing differences and similarities among the identified codes. Coding and categorizing were conducted while checking the meaning units and the transcripts to confirm the overall context. Finally, the underlying meaning that expressed the latent content of the categories was formulated into a theme (Graneheim & Lundman, 2004).

To check trustworthiness in inductive content analysis, it is suggested that one researcher be responsible for the analysis and other researchers follow up carefully on the whole process and categorization (Elo et al., 2014). In this study, the first author, who has been engaged in managing a patients’ association of breast cancer for 5 years, conducted the analysis and regular discussions were held for coding and categorizing throughout the analysis process. The third author, who has a doctoral degree in nursing and is versed in implementation and interpretation of qualitative research in cancer nursing, supervised the analysis. Peer debriefing was also conducted to review and assess the identified codes, subcategories, and categories from all interviews with the cooperation of the researchers who had experience of caring for patients with cancer. We revised the results of the analysis to achieve consensus.

### Ethical considerations

The institutional review board of Osaka University Hospital approved this study (Ref. no. 13329). Participants were provided full information about the study verbally and in writing. This included details about the purpose and procedures, the protection of confidentiality, the voluntary nature of participation, and that they could withdraw at any time without negative consequences. All participants got the opportunity to ask additional questions and gave written informed consent prior to participation. Interviews were conducted during participants’ routine treatment visits at a place in the hospital where the privacy of the participants was secured. It was emphasized that participants did not need to answer all questions because the interviews might include highly private matter. The collected data were anonymized and treated confidentially.

## Results

An identified theme of how participants perceived support in managing the HT-induced side effects provided by their partners was “Support not corresponding to transition to a new treatment.” This theme contained three categories: “Shrinking support,” “Primacy of partner,” and “Solitary new treatment.” These categories are presented in Table I.

**Table I:** Women's perceptions of support provided by their partners.

Meaning unit	Condensed meaning unit	Code	Subcategory	Category	Theme
When we go for shopping at supermarkets, my husband takes and carries shopping baskets so that I do not use my affected arm. (Participant 1)	My husband takes and carries heavy loads so that I do not use my affected arm	Partner's support related to the problems arising from past treatment	Sufficient support cultivated during past treatment	Shrinking support	Support not corresponding to transition to a new treatment
My husband does not understand the side effects I have been experiencing since the start of the hormonal therapy, although I have told him all about it. It seems that he does not think that I have changed at all because I have few observable symptoms. (Participant 7)	Although I have told my husband all about side effects, he does not understand them, most of which are unobservable	Lack of understanding of subjective side effects by the partner	Insufficient support related to the new treatment		
I do not disclose my condition to my husband. I think his mental burden is perhaps larger than mine because he kept being concerned about me for my disease [not his]. (Participant 6)	I do not disclose my condition to my husband who would feel burdened because of my cancer	Refraining from complaining about own condition	Communication with partner in a reserved attitude	Primacy of partner	
I think I should not expect too much of my husband. He continues to give me some support that he has previously done. I have to convince myself that he does support me and I cannot ask him for more support (Participant 9)	I take care not to ask my husband for more support because he has partly continued to support me like before	To moderate expectation of support from partner	Hidden expectation for partner's support		
Since the start of the hormonal therapy, I easily get offended. Trivial things that mean nothing irritate me. In contrast, sometimes I have a feeling of despair without any cause. I cannot manage my emotions. (Participant 5)	I feel more irritability and depression that are intractable for me	Huge waves of emotion	Continued distress caused by breast cancer and its treatment	Solitary new treatment	
At the start of the hormonal therapy, I assumed that it would relieve problematic side effects. However, I was confused by the hot flashes, which made me feel feverish, as if my body was burning. I have hot flashes during the day and night and these frequently disturb my sleep. These uncontrollable symptoms make me really unsettled. (Participant 4)	I was confused by the uncontrollable hot flashes because I expected that hormonal therapy would relieve problematic side effects	Side effects different from what they had expected	Suffering side effects without anticipation and understanding		
I go for shopping only for a change of scenery. Otherwise, I do not understand what I can do to manage the side effects. Unopened shopping bags go on increasing, and I feel that I have become addicted to shopping. (Participant 5)	To manage emotions, I had no choice but to shop, although it was not a good solution	Ineffective management of side effects by herself	Management of side effects using independent means		

### Shrinking support

This category showed the levels of support provided by partners. Two different levels of support were shown according to the following subcategories: “sufficient support cultivated during past treatment” and “insufficient support related to the new treatment.”

### Sufficient support cultivated during past treatments

This subcategory comprised the support that developed since the diagnosis of breast cancer until the beginning of HT and continued after starting HT. Participants received their partners’ support based on their understanding of past treatments for breast cancer. Their partners understood problems arising from past treatments and provided support related to the problems. These mainly involved protecting their upper limb on the affected side and providing financial support. Participants expressed their gratitude to their partners, as their partners generally provided good support within the range of their understanding.

Moreover, participants said that their partners considered their feelings and tried to vary the distance between them to keep them comfortable. Their partners also helped them reduce their stress and took on some responsibility of housework. Participants perceived that this behaviour newly started or improved after they were diagnosed with breast cancer and gave them the feeling that their partners treated them in a concerned and solicitous manner.

The basis of support provided by partners was marriage, giving a close relationship before treatment. Marriage allowed them to easily perceive the considerate behaviours from their partners. Participants reinterpreted the relationship they had with their partners since they got diagnosed with breast cancer. They recognized the assured affection from their partners and were confident that their partners were reliable. This gave them a feeling of security. This was represented by a comment, “My husband would be the last person to leave me in any situation” (Participant 5).

### Insufficient support related to the new treatment

Despite the good support in general, participants felt that their partners did not support them in managing side effects of HT. They said that the insufficient support stemmed from a lack of understanding by the partners. Their partners focused on observable symptoms of the side effects due to misunderstanding that the side effects included only the visible symptoms. Consequently, the partners overlooked most of the subjective symptoms experienced by the participants.

Participants could not get their partners to understand the distress caused by the side effects; therefore, there was a lack of partners’ support to manage them. Even though the partners may have noticed the invisible changes in the participants, they did not offer support to manage them because the changes were outside the range of their understanding. Participants felt that their partners thought that the treatment for breast cancer had been completed, despite the fact that they were on continuing treatment, “I think my husband has started to forget my cancer; he has been much less worried since I started HT. Maybe he thinks it is not a treatment, but just a follow-up observation” (Participant 8).

### Primacy of partner

This category showed participants’ response to insufficient support from their partners. The response was characterized into two subcategories: “communication with the partner in a reserved attitude” and “hidden expectation for partners’ support.”

### Communication with the partner in a reserved attitude

Participants were dissatisfied with the support received from their partners. However, they did not express their dissatisfaction to their partners because they recognized that they had caused their partners much trouble since they got diagnosed with breast cancer. They felt that they and their partners were not in an equal relationship, “I have been giving my husband a lot of trouble since I became ill [breast cancer]. I have changed his life” (Participant 10).

The superficial communication in the dyad made it more difficult for participants to express their desires to ask for their partners’ support. Participants and their partners did not have in-depth conversations about breast cancer and its treatment to stabilize their relationship after the diagnosis of breast cancer. Participants did not show their real feelings and kept a certain distance from their partners because, based on the superficial communication, they were unclear about what was on their partners’ minds.

### Hidden expectation for partners’ support

All participants made an effort not to increase the burden shouldered by their partners because they were afraid that their expectations of their partners would be too much. They attempted to understand their partners’ situations and moderated their expectations of support so that they could be satisfied with the existing support. Indeed, they mentally struck a balance between the past support that they considered sufficient and the current support that they considered insufficient and strained themselves to compensate for the lack of support from their partners.

Regardless, participants wished that they could share the breast cancer experience with their partners. Participants recognized that only they themselves truly understood their breast cancer experience; however, at the same time, they also expected their partners to understand. They wished their partners to understand their current experience and support them as they did during the past treatments. Participants hoped for the initiative of healthcare providers to promote their partners’ understanding.

I wish I would have received the explanation about HT with my husband when I started HT. I wish I would have shared the same contents with him. I want him to know that HT has many side effects and I feel high levels of stress even if the side effects cannot be seen from him. (Participant 5)

### Solitary new treatment

This category showed the experience of side effects in the background of insufficient support from the partners. The experience included three subcategories: “continued distress caused by breast cancer and its treatment,” “suffering side effects without anticipation and understanding,” and “management of side effects using independent means.”

### Continued distress caused by breast cancer and its treatment

Participants expressed the experience of still being affected by breast cancer and its treatment (i.e. HT). They faced physical and mental side effects. The physical side effects included hot flashes, night sweats, arthralgia, headaches, fatigue, and vaginal dryness. These symptoms limited and interfered with their activities and disturbed their usual life rhythm, which made them feel their physical condition was poor. Participants also experienced huge waves of emotion since starting HT. They felt more irascibility and irritability, and felt extreme depression. Depressive mood was sometimes described as a feeling of despair. The wave of emotion was intractable for them and affected their composure.

My emotional condition has changed since starting HT. It deprived me of my vitality. My mind is getting thinner and weaker. Concurrently, I have certainly become [more] short-tempered than before. I am easily irritated and grow angry immediately, to anybody else, everywhere. (Participant 4)

In addition, as HT was a persistent reminder of breast cancer, it resulted in an ongoing experience of breast cancer for participants. Every time they took daily HT medication, they were reminded that they had breast cancer. Therefore, they spent a lot of time considering the fact that they suffered from breast cancer, and some participants still had not accepted this. Participants continued to experience the emotional distress caused by breast cancer. They talked about their constant anxiety regarding recurrence and metastasis. They felt strongly that no treatment could completely prevent recurrence or metastasis. When they perceived their physical condition changing, their anxiety about disease progression increased even when they felt only slightly different.

### Suffering side effects without anticipation and understanding

Participants described HT-related side effects as unexpected and vague symptoms. They expressed dissatisfaction with the explanation that they received at the start of HT because treatment commenced without them fully understanding HT and its side effects. The lack of information led to an assumption that HT would relieve problematic side effects, and this optimistic assumption made the experience of side effects different from what they had expected. Participants were perplexed by the unexpected symptoms and were unsettled that the symptoms were beyond their expectations, “I expected that I would recover my health smoothly when surgery ended. Contrary to expectation, I am bothered [by] plural symptoms since starting HT. I feel uneasy because I am not still restored to health” (Participant 6).

Although they felt that their condition had certainly become poor since starting HT, it was difficult to separate the HT-induced symptoms experienced from symptoms induced by other causes, including ageing, past treatment for breast cancer, and comorbidity. Moreover, most of the HT-related side effects were strongly subjective, which increased the difficulty in understanding the impact of HT.

### Management of side effects using independent means

This subcategory represented participants’ own effort to manage the side effects in the lack of support from partners. Participants sought to manage side effects with self-known techniques although they experienced difficulty in managing them on their own. To buffer the effect of physical symptoms, they repeatedly tried some techniques they thought of. The waves of emotion were more difficult to manage and were dealt with passively.

Increased difficulty in managing the side effects sometimes resulted in using avoidance as a strategy to deal with them. To keep their composure, distraction and cognitive avoidance were used, “I feel like I have not taken the side effects seriously. I try not to keep them in mind. At least, that is what I aim to do. If not, I would be constantly troubled with them” (Participant 10).

## Discussion

Participants experienced the decline in support from the partners. Partners’ support was shrinking and support related to HT as a new treatment was insufficient. However, the participants gave priority to their partners and aimed to compromise the support that partners currently provided. They were left in a continuing trajectory of breast cancer and experienced solitary new treatment.

### A new phase of breast cancer treatment in which less understanding and support was provided by partners

The results of this study suggest that, after starting HT, women enter a new treatment phase in which their partners do not show as much understanding. Although participants recognized that their partners had supported them proactively in general, they sensed a gap between the distress they experienced from their side effects and their partners’ perception; they felt that their partners did not understand their experience of the side effects. This result is a distinguishing feature of the experience of receiving HT. Indeed, it contradicts previous reports that family caregivers and significant others overestimate the symptoms rather than underestimate them when compared with the patients themselves (Carlson et al., 2001; Lobchuk & Degner, 2002; McMillan & Moody, 2003; Sneeuw, Sprangers, & Aaronson, 2002; Yeşilbalkan & Okgün, 2009).

The characteristics of HT and its side effects appear to contribute to the lack of understanding by partners. Treatments such as surgery and chemotherapy are easily understood to be highly invasive treatments regardless of levels of knowledge the partners have. However, most of the side effects induced by HT cannot be observed and are highly subjective, meaning that partners require knowledge to understand them. Previous studies have reported poorer agreement between patients and family caregivers when comparing the assessment of subjective psychological symptoms against objective physical symptoms (Lobchuk & Degner, 2002; Yeşilbalkan & Okgün, 2009). HT affected both the physical and psychological health of the participants, causing them to experience a wave of emotions that their partners often failed to notice. In addition, when receiving HT, women are treated mainly with oral medications as outpatients, which could lead to the erroneous assumption that HT has few side effects and that women have completed their treatment.

Poor understanding by participants could also exacerbate the lack of understanding by their partners. Participants could not explain the symptoms, interpret their experience, or evaluate the impact of HT, as the side effects of HT were unexpected and vague. This seemed to make it more difficult for the partners to understand the side effects experienced by participants receiving HT. However, this poor understanding among participants partially resulted from a lack of information, as supported by a recent study in which women receiving HT were shown to feel dissatisfaction with the information provided for symptom management from healthcare providers (Van Londen et al., 2014). For patients with chronic disease and symptoms overwhelming daily life, adequate knowledge and understanding help them live with their disease and deal with the symptoms (Bennion & Molassiotis, 2013; Knobf, 2013; Lidén, Björk-Brämberg, & Svensson, 2015; Nordgren, Asp, & Fagerberg, 2010). Women receiving HT also have a need to prepare for side effects.

### The need to educate partners

Insufficient support related to HT resulted from the lack of understanding by couples as stated above. To maintain the same level of support as that provided during past treatment, partners as well as women need to understand HT and its side effects. Participants hoped for educational support to promote their partners’ understanding. Partners’ support has the potential to improve the management of HT-related side effects. Several studies suggest that support from partners can improve psychological well-being and promote adaptation among patients with cancer (Bloom et al., 2001; Brandão et al., 2014; Carver et al., 2006; Pistrang & Barker, 1995; Regan et al., 2012). These effects would also be beneficial for women receiving HT who face difficulties managing the effect on their emotions and the anxiety about recurrence and metastasis. Educational support for the partners could improve the partners’ understanding of the subjective distress experienced by women. Ultimately, this could help women manage their emotions and decrease their distress.

Educational support for partners could also promote voluntary support for women because the lack of support provided to manage the side effects was assumed to result from limited knowledge and understanding among partners. Although the participants recalled the support that their partners provided during earlier treatment and compromised on receiving existing support, the relative lack of support during the current treatment might increase the risk of depression, poorer quality of life, and negative adaptation to the experience (Douglass, 1997; Knobf, 2008; Manne, Ostroff, Winkel, Grana, & Fox, 2005). Participants often hesitated to ask for support as they cared about their partners and appreciated their continuous support. Thus, educational support should focus on encouraging partners to offer voluntary support.

### Limitations

We only recruited women who recognized their partners as the main source of social support, which could limit the experience of women who receive support from their partners. In addition, it is required to explore the experiences of women receiving HT with larger number of samples. Another limitation is that we only explored the experience from the women's perspective. Further studies are needed to ensure a comprehensive understanding of the experience of couples during HT.

### Implications for nursing

Educational support for couples would improve the lack of understanding shown by women and their partners, enabling them to share subjective symptoms that were not obvious to partners and manage them together. Moreover, nurses must avoid minimizing the side effects of HT, which can be numerous, highly subjective, and difficult to manage. Improving symptom management of side effects remains important during HT. Enhancing the support provided by partners may be the key to improved management. At the same time, nurses must continue to provide information about other sources of support and strategies to relieve the side effects of treatment.

## Conclusions

Support given by the partners during HT does not correspond to transition to a new treatment. Although women receiving HT experience significant psychological and physical symptoms and are still in the continuing trajectory of breast cancer, the partners are unable to support women. The lack of support from the partners results from their little understanding of the subjective side effects that are often not obvious to them. Support from partners can help to improve the psychological well-being of women receiving HT. Therefore, nurses need to provide educational support that is directed at helping couples share the side effects and manage them together.
